# TIPE Family of Proteins and Its Implications in Different Chronic Diseases

**DOI:** 10.3390/ijms19102974

**Published:** 2018-09-29

**Authors:** Devivasha Bordoloi, Kishore Banik, Bano Shabnam, Ganesan Padmavathi, Javadi Monisha, Frank Arfuso, Arunasalam Dharmarajan, Xinliang Mao, Lina H. K. Lim, Lingzhi Wang, Lu Fan, Kam Man Hui, Alan Prem Kumar, Gautam Sethi, Ajaikumar B. Kunnumakkara

**Affiliations:** 1 Cancer Biology Laboratory, DBT-AIST International Laboratory for Advanced Biomedicine (DAILAB), Department of Biosciences and Bioengineering, Indian Institute of Technology Guwahati, Assam 781039, India; devivasha@iitg.ac.in (D.B.); kishore.banik@iitg.ac.in (K.B.); bano176106104@iitg.ac.in (B.S.); padmavathi@iitg.ac.in (G.P.); j.monisha@iitg.ac.in (J.M.); 2 Stem Cell and Cancer Biology Laboratory, School of Pharmacy and Biomedical Sciences, Curtin Health Innovation Research Institute, Curtin University, Perth, WA 6009, Australia; frank.arfuso@curtin.edu.au (F.A.); a.dharmarajan@curtin.edu.au (A.D.); 3 Institute of Clinical Pharmacology, Guangzhou University of Chinese Medicine, Guangzhou 510405, China; xinliangmao@suda.edu.cn; 4 Department of Pharmacology, College of Pharmaceutical Sciences, Soochow University, Suzhou 215123, China; 5 Department of Physiology, Yong Loo Lin School of Medicine, National University of Singapore, Singapore 117456, Singapore; lina_lim@nuhs.edu.sg; 6 NUS Immunology Program, Life Sciences Institute, Centre for Life Sciences, National University of Singapore, Singapore 117456, Singapore; 7 Department of Pharmacology, Yong Loo Lin School of Medicine, National University of Singapore, Singapore 117600, Singapore; csiwl@nus.edu.sg (L.W.); phcfanl@nus.edu.sg (L.F.); csiapk@nus.edu.sg (A.P.K.); 8 Cancer Science Institute of Singapore, National University of Singapore, Singapore 117599, Singapore; 9 Division of Cellular and Molecular Research, Humphrey Oei Institute of Cancer Research, National Cancer Centre, Singapore 169610, Singapore; cmrhkm@nccs.com.sg; 10 Medical Science Cluster, Yong Loo Lin School of Medicine, National University of Singapore, Singapore 119077, Singapore; 11 Curtin Medical School, Faculty of Health Sciences, Curtin University, Perth, WA 6009, Australia

**Keywords:** TIPE, TIPE1, TIPE2, TIPE3, chronic diseases, cancer

## Abstract

The tumor necrosis factor-α-induced protein 8-like (TIPE/TNFAIP8) family is a recently identified family of proteins that is strongly associated with the regulation of immunity and tumorigenesis. This family is comprised of four members, namely, tumor necrosis factor-α-induced protein 8 (TIPE/TNFAIP8), tumor necrosis factor-α-induced protein 8-like 1 (TIPE1/TNFAIP8L1), tumor necrosis factor-α-induced protein 8-like 2 (TIPE2/TNFAIP8L2), and tumor necrosis factor-α-induced protein 8-like 3 (TIPE3/TNFAIP8L3). Although the proteins of this family were initially described as regulators of tumorigenesis, inflammation, and cell death, they are also found to be involved in the regulation of autophagy and the transfer of lipid secondary messengers, besides contributing to immune function and homeostasis. Interestingly, despite the existence of a significant sequence homology among the four members of this family, they are involved in different biological activities and also exhibit remarkable variability of expression. Furthermore, this family of proteins is highly deregulated in different human cancers and various chronic diseases. This review summarizes the vivid role of the TIPE family of proteins and its association with various signaling cascades in diverse chronic diseases.

## 1. Introduction

The tumor necrosis factor-α-induced protein 8 (TIPE/TNFAIP8/Oxi-α) family is a newly identified family of proteins that is involved in the regulation of immunity and tumorigenesis. This family is comprised of four members, namely, TIPE or TNFAIP8 (tumor necrosis factor-α-induced protein 8), TIPE1 (TNFAIP8-like 1, or TNFAIP8L1), TIPE2 (TNFAIP8-like 2 or TNFAIP8L2), and TIPE3 (TNFAIP8-like 3 or TNFAIP8L3) [1]. Despite the fact that all the four proteins of this family share a significant sequence homology (~54% sequence identity), they are involved in the regulation of different cellular activities [2,3,4,5].

TIPE, the most extensively studied member of this family, is a transcription factor nuclear factor- κ-B inducible, anti-apoptotic, and oncogenic molecule that is associated with prognosis of different malignancies. It is a 21-kDa cytoplasmic protein that was initially identified in human head and neck squamous cell carcinoma [5,6,7,8,9,10]. It is expressed in different human normal tissues with relatively higher levels in placenta and lymphoid tissues. The open reading frame of this protein bears a sequence in the amino terminus that displays a notable homology to the death effector domain II of the cell death regulatory protein, Fas-associated death domain-like interleukin-1β-converting enzyme-inhibitory protein (FLIP) [11]. TIPE is associated with the immune regulation of CD4^+^ T lymphocytes and inhibits autophagy under oxidative stress through the mammalian target of rapamycin (mTOR)-dependent pathway [3,12,13]. Notably, different transcript variants of this TIPE gene were recently listed in the NCBI databank. However, currently no study has described their distinguished roles or depicted the factors that regulate their expression. A study by Lowe and group reported TIPE variant 2 as an oncogenic gene product that may regulate different processes in tumor cells such as proliferative signaling, resistance to cell death, and evasion of growth suppressors. On the other hand, other variants are normally downregulated in cancer (variant 1) or show minimal expression in cancer or normal tissues (variant 3–variant 6) [14].

TIPE1 (tumor necrosis factor-α-induced protein 8-like 1) is a recently identified member of the TIPE family that can act as a cell death regulator. It is regarded as a pro-apoptotic factor with the ability to cause increased apoptotic functions. Currently, there is little information available about the role of TIPE1. The information on its biological activity under both physiological and pathological conditions remains ambiguous [4,5,15,16,17]. It was reported to be distributed in different mouse tissues except for mature B and T lymphocytes. Further, TIPE1 was speculated to be associated with cardiac decompensation linked with diabetes and to interact with FBXW5 and caspase-8. Besides, different post-translational modifications were also predicted to exist in the case of TIPE1 [2,16].

TIPE2 (tumor necrosis factor-α-induced protein 8-like 2), the third member of this family, is a latterly discovered negative regulator of innate, as well as cellular immunity, with sizable sequence homology with the other members of the family [18,19,20]. It is a cytoplasmic protein consisting of 184 amino acids and is expressed preferentially in lymphoid tissues and some non-lymphoid tissues [19,21]. This protein was initially identified as an abnormally expressed gene in the inflamed spinal cord of experimental autoimmune encephalomyelitic mice [22,23]. Further, TIPE2 was found to be expressed in varied cell types such as neurons in the brain and brainstem; hepatocytes; squamous epithelial cells in the cervix and esophagus; glandular epithelial cells in the colon, stomach, and appendix; and transitional epithelial cells in the ureter and bladder [24]. It negatively regulates the functions of toll-like receptor (TLR) and T cell receptor, and its selective expression in the immune system averts hyper responsiveness and maintains immune homeostasis [22,23,25]. Further, it is an inhibitor of the nuclear factor κ-light-chain-enhancer of activated B cells (NF-κB) and mitogen-activated protein kinase (MAPK) signaling pathways and contributes to the reduced activation of activator protein-1 (AP-1) and NF-κB [5,26,27]. It also acts as an inhibitor of Rac, which is a GTPase involved in the promotion of trailing-edge polarization [28]. A recent genome-wide expression profiling analysis reveals TIPE2 to function as an immune checkpoint regulator of inflammation and metabolism. This finding depicts that during the course of inflammation, the expression of TIPE2 may be downregulated plausibly due to its altered epigenetic status, which in turn results in the upregulation of the expression of lipid biosynthesis genes, mitochondrial respiration, and inflammation [29].

TIPE3 (tumor necrosis factor-α-induced protein 8-like 3), the newest member of TIPE family, is located on human chromosome 15. It functions as a transfer protein for lipid second messengers PIP2 (phosphatidylinositol 4,5-bisphosphate) and PIP3 (phosphatidylinositol 3,4,5-trisphosphate), and enhances their level in the plasma membrane [1,30,31]. This protein is expressed in various human organs and is highly upregulated in several human cancers such as cervical cancer, colon cancer, esophageal cancer, and lung cancer [5,32].

Furthermore, the crystal structures of two members of the TIPE family, namely, TIPE2 and TIPE3 from *Homo sapiens*, have been determined. Both of them possess a central hydrophobic cavity that is proposed as a binding site for cofactors, occupied by two long electrostatic densities, which are plausibly phospholipid in nature [3,19,33,34]. Moreover, these phospholipids were observed to share similar binding modes that involve the exposure of the inositol head group outside and insertion of the lipid tails into the cavity. In addition, all the lipid molecules interact with the critical, positively charged residues as per molecular interaction studies, i.e., Arg 75 and −91 in TIPE2, and Arg 181 and 197 in TIPE3, indicating the similar binding fashion of the phosphoinositides to the TH domain of this protein family [35]. Interestingly, the high-resolution crystal structure of TIPE2 clearly reveals that it possesses a unique yet previously uncharacterized fold that gives TIPE2 a unique structure and topology that is different from that of death effector domain (DED). The structure of TIPE2, which comprises around 150 amino acids, is reasonably larger than that of the DED, as it usually contains a total of 90 amino acids. Again, the topology of TIPE2 is different from that of a DED, as it was observed that N-to-C arrangement of TIPE2 is identical to the C-to-N topology diagram of DED. Therefore, the topology of TIPE2 seems to be a mirror image of that of the DED [34]. Additionally, the crystal structure of TIPE from *Mus musculus* (mTIPE) was also determined. The overall shape of mTIPE bears a resemblance to a water dipper. Its cylindrical domain contains two long electron densities and has a dimension of 48 × 31 × 30 Å linked to an N-terminal grip-like domain of length ~35 Å that comprises of 20 residues. It possesses a hydrophobic cavity of depth around 20 Å, a diameter of around 7 Å, and a volume of 837 Å, which is lined with highly conserved hydrophobic residues, thereby facilitating the binding of hydrophobic cofactors or substrates inside the cavity [3].

**Figure 1:**
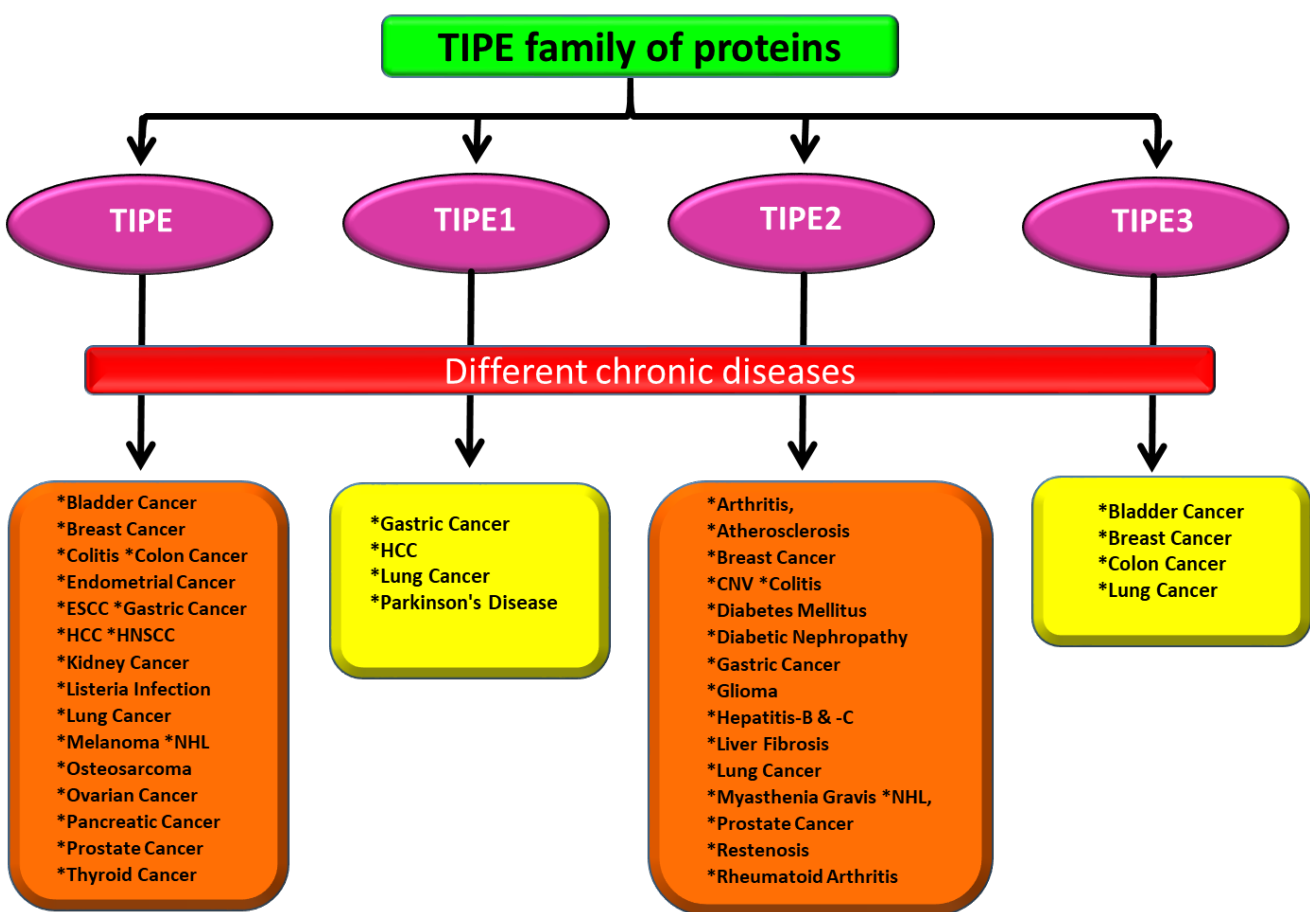
Various chronic diseases associated with TIPE family of proteins.

Aforementioned, different *in vitro* and *in vivo* studies have revealed that this family of proteins plays a crucial role in inflammatory responses and tumorigenesis (Table 1; [5]). Interestingly, the expression analyses in clinical settings have also demonstrated these proteins to be highly deregulated in different cancers and various chronic diseases (Figure 1).

This review summarizes the role of this TIPE family of proteins in different chronic diseases, their molecular targets and associated signaling cascades in different chronic diseases based on existing literature.

## 2. Role of TIPE Family of Proteins in Different Chronic Diseases

### 2.1. TIPE Family of Proteins and Cancers

Cancer, which stems from the perturbations of multiple signaling pathways, affects people of all ages and is a major health concern worldwide [5,91,92]. The TIPE family of proteins plays a vital role in carcinogenesis and metastasis through its deregulated expression and function. It has been found to be strongly associated with cancers of breast, bone, brain, cervix, colon, esophagus, endometrium, liver, lung, stomach, and thyroid. Overall, the potential crosstalk of the four different TIPE proteins with various signal transduction cascades in different cancers has been reviewed by our group [5] previously.

### 2.2. TIPE Family of Proteins and Inflammatory Diseases

TIPE and TIPE2, the regulators of immunity, have been demonstrated to protect against inflammatory diseases such as atherosclerosis, colitis, and rheumatoid arthritis.

#### 2.2.1. Atherosclerosis

**Figure 2:**
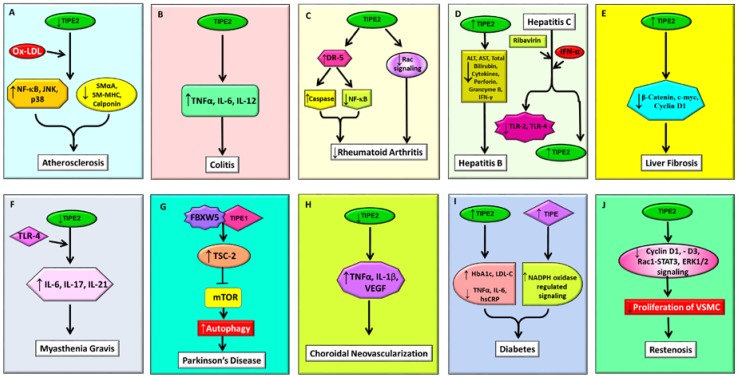
Role of TIPE family of proteins and their molecular mechanisms in different chronic diseases; (**A**). Atherosclerosis, (**B**). Colitis, (**C**). Rheumatoid Arthritis, (**D**). Hepatitis B and C, (**E**). Liver Fibrosis, (**F**). Myasthenia Gravis, (**G**). Parkinson’s Disease, (**H**). Choroidal Neovascularization, (**I**). Diabetes, (**J**). Restenosis

Atherosclerosis is widely known as an inflammatory disease of the arterial wall in which macrophages play an important role. Notably, TIPE2 exhibits a high expression level in resting macrophages and has been found to exhibit a potent atheroprotective role by regulating macrophage responses to oxidized low-density lipoprotein (ox-LDL). When macrophages lacking TIPE2 were treated with ox-LDL, it resulted in enhanced production of oxidative stress and pro-inflammatory cytokines, as well as activation of NF-κB, JNK, and p38 signaling cascades. These results clearly implied TIPE2, a new-found inhibitor of atherosclerosis, to be an effective target against this disease [71]. Further, TIPE2 displayed its atheroprotective role through modulation of phenotypic switching of vascular smooth muscle cells (VSMCs), which plays a vital role in the development of atherosclerosis in response to ox-LDL stimuli. Ox-LDL treated TIPE2-deficient VSMCs were found to have lower expression of contractile proteins such as smooth muscle-myosin heavy chain (SM-MHC), smooth muscle α-actin (SmαA), and calponin, while proliferation, migration, and the synthetic ability for cytokines and growth factors were found to increase significantly [72] (Figure 2A). Thus, these findings clearly imply that TIPE2 is an atheroprotective protein that may serve as a potent drug candidate for protection against this inflammatory disease.

#### 2.2.2. Colitis

TIPE2 plays a vital role in inflammatory cell function and commensal bacteria dissemination regulation in dextran sodium sulfate (DSS)-induced colitis. Lou and group observed that mice with TIPE2 deficiency in the hematopoietic compartment survived longer compared to the wild types upon treatment with DSS. Further, it was observed that the degree of severity of colitis, as well as colonic damage in TIPE2-deficient mice, was notably less and was plausibly attributed to the decreased colonic expression of inflammatory cytokines TNF-α, interleukin (IL)-6, and IL-12. In addition, it was observed that TIPE2-deficient mice with ameliorated DSS-induced colitis also displayed a weaker systemic inflammatory response together with reduced local dissemination of commensal bacteria [73]. Another study investigated the role of TIPE in DSS-induced colitis in which TIPE-deficient mice were reported to be more prone to DSS-induced colitis, and that lack of expression of TIPE in non-hematopoietic cells was found to play a vital role. In TIPE knockout mice, a great reduction in body weight, the occurrence of severe diarrhea, rectal bleeding, and increased mortality was observed, exemplifying the role of TIPE in protection against DSS-induced colitis [74] (Figure 2B). Altogether, these two findings indicate that both TIPE and TIPE2 play important roles in the maintenance of colon homeostasis and the prevention and treatment of colitis. However, further in-depth studies are required to clearly understand the exact molecular mechanism(s) of actions of these proteins against colitis.

#### 2.2.3. Rheumatoid Arthritis

Rheumatoid arthritis is a chronic inflammatory illness characterized by joint tenderness, joint swelling, and synovial joint destruction, resulting in severe disability and premature mortality [93]. Fibroblast-like synoviocytes (FLSs) play an important role in the pathology of rheumatoid arthritis. The study conducted by Shi and group indicated that TIPE2 increased adjuvant arthritis (AA)-FLSs apoptosis through enhanced DR5 expression levels, thus inhibiting NF-κB activation and promoting the activation of caspase in AA-FLSs. [75]. Further, TIPE2 was found to regulate lipopolysaccharide-induced rat rheumatoid arthritis immune responses via activation of Rac and phosphorylation of interferon regulatory factor 3. This study depicted TIPE2 to be inversely associated with cytokine gene expression in synovial fibroblasts after lipopolysaccharide stimulation. Thus, TIPE2 plays a negative role in the activation of the Rac signaling pathway, as well as initiation of the immune response via reduced function of pro-inflammatory cytokines [76] (Figure 2C). Thus, using this novel target, TIPE2, therapeutic strategies against rheumatoid arthritis can be designed and used for protection against this disease.

### 2.3. TIPE Family of Proteins and Infectious Diseases

Various studies were performed to evaluate the association of the TIPE family of proteins and different infectious diseases such as hepatitis B, hepatitis C, listeria infection, and liver fibrosis.

#### 2.3.1. Hepatitis B

Hepatitis B virus (HBV)-induced hepatic inflammation affects a vast majority of people across the world and is also recognized as a prime cause of hepatic cancer. It has been reported that TIPE2, a regulator of immune receptor signaling, can control HBV-induced hepatitis. Xi and colleagues reported that patients with chronic hepatitis B exhibited remarkably decreased TIPE2 expression in their peripheral blood mononuclear cells (PBMCs) compared to healthy individuals. Further, the expression of TIPE2 negatively correlated with the blood levels of aspartate aminotransferase (AST), alanine aminotransferase (ALT), total bilirubin, and the HBV load of the patients, suggesting that TIPE2 is an important marker in HBV-induced hepatic inflammation [79]. In addition, the expression of TIPE2 was found to be relatively higher in acute-on-chronic hepatitis B liver failure (ACHBLF) patients compared to healthy controls, which positively correlated with total serum bilirubin, international normalized ratio, and the model for end-stage liver disease scores. Additionally, the TIPE2 mRNA level was significantly higher in non-survivors compared to survivors in patients with ACHBLF, and the TIPE2 mRNA level was found to be reduced progressively in survivors together with signs of recovery from patients with ACHBLF. Further, lipopolysaccharide stimulation in ACHBLF patients resulted in reduced levels of IL-6, as well as TNF-α, which displayed a negative association with TIPE2 [80]. Another study investigated the expression of TIPE2 in mice PBMCs with autoimmune hepatitis (AIH) and its involvement in the pathogenesis of AIH. The results showed that TIPE2 was expressed less in AIH mice, whereas in the case of concanavalin A-induced AIH, TIPE2-deficient mice exhibited enhanced levels of serum ALT, AST, pro-inflammatory cytokines, and severe hepatic inflammation [77]. Zhang and group reported that the TIPE2 protein level in PBMCs of hepatitis B patients was significantly less and negatively associated with the aminotransferases sera values. Notably, CD8^+^T cells, which express a low level of TIPE2, produced significantly high granzyme B, perforin, and interferon-γ, resulting in their enhanced cytolytic effect [78]. Further, in chronic hepatitis B (CHB) patients, TIPE2 mRNA level in immune clearance phases was notably more compared to the immune tolerance phase, indicating that TIPE2 might be involved in immune clearance of patients with CHB. In addition, TNF-α, interferon-γ, and HBV DNA load were also observed to be independently linked with the level of TIPE2 in CHB patients [81] (Figure 2D).

#### 2.3.2. Hepatitis C

Approximately 80% of chronic hepatitis cases are caused due to infection with Hepatitis C virus (HCV). TIPE2 has been found to play an important role in chronic hepatitis C (CHC) infection. Kong et al. showed that in CHC patients, TIPE2 gets significantly downregulated, whereas TLR2 and TLR4 show upregulation when compared to healthy controls. Further, the mRNA expression level of TIPE2 was found to be negatively associated with serum ALT, AST, and HCV RNA levels, as well as TLR2 and TLR4 mRNA levels in CHC patients. In addition, treatment of HCV patients with ribavirin and interferon-α led to the upregulation of TIPE2 mRNA and downregulation of TLR2 and TLR4 mRNA level [82] (Figure 2D).

Taken together, TIPE2 possesses a strong correlation with the infection with hepatitis virus, and hence it can used as a target to develop strategies for the management of hepatitis-infected patients.

#### 2.3.3. Listeria Infection

TIPE was reported to regulate infection with *Listeria monocytogenes* by controlling pathogen invasion and host cell apoptosis in a Rac1 GTPase-dependent manner. Notably, TIPE-knockout mice were found to be resistant to lethal *Listeria monocytogenes* infection, and they exerted a decreased bacterial load in the liver and spleen. In addition, knockdown of TIPE in murine liver cells resulted in enhanced apoptosis, reduction in bacterial invasion into cells, and deregulated Rac1 activation [83]. These findings provide understanding towards the role of TIPE2 in the pathogenesis of listeria infection, and thus it can be used as a therapeutic target for listeriosis.

#### 2.3.4. Liver Fibrosis

TIPE2 possesses a protective effect on liver fibrosis and hence may serve as a potent target against this disease. TIPE2 diminished liver fibrosis through reversal of activated hepatic stellate cells. Xu et al. demonstrated low expression of TIPE2 in CCl4-treated murine primary HSCs and activated HSC-T6 cells. Overexpression of TIPE2 hindered the activation and proliferation of HSC-T6 cells, as well as the expression of c-myc, cyclin D1, and β-catenin, whereas its inhibition displayed the reverse effect [84] (Figure 2E). Thus, owing to its protective effect, TIPE2 displays potential as an effective target against liver fibrosis.

### 2.4. TIPE Family of Proteins in Neuromuscular and Neurodegenerative Diseases

TIPE2 and TIPE1 have been found to exert their effect in Myasthenia Gravis and Parkinson’s disease, respectively, and thus can serve as important targets for therapies against these diseases.

#### 2.4.1. Myasthenia Gravis

Myasthenia gravis (MG) is an autoimmune neuromuscular disease, the incidence of which is increasing. TIPE2 has been found to play role in MG via modulation of autoimmune T helper 17 cell responses mediated by TLR4 [85]. The study showed downregulation of TIPE2 in MG compared to normal controls. Furthermore, TIPE2 exerted a negative association with the levels of IL-6, -17, and -21 in the serum of MG patients. In cultured MG PBMC, TLR4 activation caused downregulation of TIPE2, whereas RORγt expression and IL-6, -17, -21 production was enhanced. Nevertheless, overexpression of TIPE2 abrogated the TLR4 activation-induced effects [85] (Figure 2F). Collectively, this study provides evidence that targeting TIPE2, which functions as a negative regulator of immunity, may offer protection against this autoimmune disease.

#### 2.4.2. Parkinson’s Disease

Parkinson’s disease is a long-term degenerative disorder that involves the central nervous system. Altered regulation of TIPE1 may contribute to deregulated autophagy seen in dopaminergic neurons under pathogenic oxidative stress, which is especially observed in post-mortem brains in Parkinson’s disease. This protein binds to FBXW5, a tuberous sclerosis complex 2 (TSC2; a negative regulator of mTOR) binding receptor present in CUL4 E3 ligase complex, resulting in enhanced autophagy via activation of TSC2 in a Parkinson’s disease model. Further, oxidative, stress-induced TIPE1 caused stabilization of TSC2 protein, reduction in mTOR phosphorylation, and an increase in autophagy [86]. Another study conducted by Kouchaki and group attempted to evaluate the association between the serum levels and circulatory gene expression of TIPE2 with severity of Parkinson’s disease by enrolling a total of 43 patients. The results implied that no significant differences were noted between the mean serum levels and TIPE2 expression in patients compared to the healthy individuals. They further showed that enhanced serum levels of TIPE2 possess a direct correlation with age and severity of patients with Parkinson’s diseases. Besides, TIPE2 expression was also found to be strongly linked with the age of the patients [94] (Figure 2G).

### 2.5. The TIPE Family of Proteins and Other Chronic Diseases

Apart from the abovementioned, this newly discovered family of proteins is strongly involved in various other diseases such as choroidal neovascularization, restenosis, and metabolic disease-like diabetes.

#### 2.5.1. Choroidal Neovascularization (CNV)

Choroidal neovascularization (CNV), a pathological condition commonly occurring in ocular diseases, is primarily characterized by vasculogenesis and angiogenesis of the neuroretina, with the retinal pigment epithelium (RPE) as a major target. TIPE2, a negative regulator of immunity, has been found to play a role in CNV, as inflammation and immunity are critical in the early development of CNV. Suo and group conducted a study that reported that TIPE2 is present in human RPE cells in the cytoplasm, as well as the nucleus, and was downregulated in the inflammatory condition, with a subsequent reduction in cell viability. Further, knock-down of TIPE2 resulted in the upregulation of TNF-α, IL-1β, and VEGF, especially under lipopolysaccharide induced stimuli. As TIPE2 displays potent anti-angiogenic properties and VEGF plays a vital role in the final stage of neovascularization, TIPE2 might take part in CNV formation [87] (Figure 2H). However, comprehensive studies are vital to decipher the underlined molecular mechanisms through which TIPE2 function and help in the development of CNV.

#### 2.5.2. Diabetes

Diabetes is a type of metabolic disease associated with high blood sugar levels. Although TIPE2 plays a key role in inflammatory homeostasis, its exact role in type 2 diabetes mellitus (T2DM) remains unknown. Liu and group reported that TIPE2 is involved in T2DM via modulation of TNF-*α*. They observed an increased level of TIPE2 in T2DM patients that was positively associated with hemoglobin A1c and low-density lipoprotein cholesterol, while it negatively correlated with serum TNF-*α*, IL-6, and hsCRP concentrations in the diabetic patients. Further, treatment with high glucose concentrations resulted in the upregulation of TIPE2 and cytokine secretion in differentiated THP-1 human monocyte cells. Additionally, TIPE2 adenovirus infection reversed the enhanced TNF-*α* level, whereas treatment with siTIPE2 aggravated the enhanced level of TNF-*α* and IL-6 in differentiated THP-1 cells under high glucose conditions [88]. Again, TIPE serves as a vital component of a signaling cascade linking mesangial cell proliferation and diabetic renal injury. The study conducted by Zhang and colleagues showed that in response to high glucose, TIPE was upregulated in mesangial cells, and the expression of TIPE was directly correlated with mesangial cell proliferation mediated via an NADPH oxidase-regulated signaling pathway [89] (Figure 2I).

The above studies illustrated the critical role of the two TIPE family of proteins, namely, TIPE and TIPE2 in diabetes and diabetic nephropathy. Hence, they may serve as effective targets against diabetes mellitus and may aid in the development of therapeutic strategies for the prevention and treatment of this metabolic disorder.

#### 2.5.3. Restenosis

Restenosis is a disease characterized by smooth muscle cell hyperplasia and neointimal formation. Zhang and group reported that TIPE2 repressed injury-induced restenosis through inhibited proliferation of vascular smooth muscle cells (VSMCs) via modulation of ERK1/2 and Rac1-STAT3 signaling cascades. The study reported that enforced TIPE2 expression suppressed the proliferation and blocked cell cycle progression in VSMCs, while deficiency of TIPE2 induced proliferation of VSMCs and upregulation of cyclin D1 and cyclin D3 [90] (Figure 2J). Therefore, targeting TIPE2 might help in designing novel approaches against restenosis.

Altogether, this family is evinced to have profound role in different chronic diseases. Interestingly, the function of TIPE and TIPE2 in different chronic diseases and their mode of action have been studied extensively. However, focus needs to be given to unveil the role of TIPE in various chronic diseases other than cancer. In addition, much comprehensive studies are immensely critical to elucidate the roles of the other two members of this family of proteins such as TIPE1 and TIPE3 in the development of different chronic diseases and to unveil their underlined molecular mechanisms. This would help us not only to understand their exact functions, but also to develop novel therapeutic approaches for the prevention and treatment of diverse chronic diseases effectively.

## 3. Conclusions

The TIPE protein family presents a novel group of proteins discovered just a decade ago. Expression studies of these proteins show remarkable variability among themselves. Interestingly, although the proteins of this family were initially depicted as the modulators of tumorigenesis, inflammation, and cell death, they were also found to possess various other functions. For instance, TIPE and TIPE1 function as autophagy inhibitors and activators in experimental models of Parkinson’s disease. Further, they are involved in the transfer of lipid secondary messengers PIP2 and PIP3. Members of the TIPE family have been associated with the regulation of immune function and homeostasis and the development of diverse cancer types. Most importantly, the members of this family share a significant sequence homology but are involved in different biological activities. For instance, TIPE1 exhibits a high degree of sequence homology with TIPE2. Despite the existence of a common fold, TIPE2 plays a vital role in immune homeostasis, whereas TIPE1 may not play an essential role in immunity. Despite the existing knowledge on this protein family in the literature, a lot more needs to be elucidated. Though this family of proteins plays an important role in carcinogenesis, metastasis, and development of different human chronic diseases either through up- or downregulation, its exact molecular functions, detailed mechanism of action, and the plausible crosstalk between its members remain ambiguous. Therefore, more comprehensive studies are imperative for better understanding of this important family of proteins, which would provide key insights for biomarker discovery and treatment strategies for a wide array of chronic diseases.

## Figures and Tables

**Table 1:** Different chronic diseases and signaling pathways with known associations with the TIPE family of proteins.

Disease	Model	Protein Involved	Targets Associated/Outcome	References
**Cancers**				
Bladder cancer	T24 cells	↑TIPE3	↑PI3K-Akt, ↑MEK-ERK	[31]
Breast cancer	Tissue samples	↑TIPE	tumor progression	[8]
	MDA-MB-231 and MCF-7 cells	↑TIPE2	↓β-catenin, ↓cyclin D1, ↓c-Myc	[36]
	MDA-MB-231 cells transfected into the dorsal flank of female BALB/c nude mice (4–5 weeks old); two groups (vector group and TIPE2 group)		↓tumor growth	
	MCF-7 cells	↓TIPE	↑p21	[14]
	MDA-MB-231 and LM2-4175 cells	↓TIPE	↑SNX1, ↑NR4A1, ↑AP2A1, ↓IL5 ↓SRC, ↓MAPT, ↓NEK2, ↓TRAF4, ↓PDCL, ↓GTF2F2, ↓GRAP2, ↓ABL1, ↓AKAP2, ↓GAP43, ↓PIK3CA, ↓EGFR,	[9]
	HS578T and MCF-7 cells	TIPE	-	[37]
	MDA-MB-435 cells MDA-MB 435 cells transfected via tail vein into female BALB/c mice (6–8 weeks old); eight animals in vector group and 12 animals in TIPE group	↓TIPE	↓VEGFR-2, ↓MMP-1, ↓MMP-9	[38]
	MCF-7 and MDA-MB-231	TIPE3	↑MMP2, ↑uPA, ↑AKT, ↑NF-κB	[32]
	MDA-MB-231 cells	↑TIPE	-	[39]
Cervical cancer	Tissue samples	↑TIPE	platinum resistance	[40]
Colon cancer	CACO2 and HCT116 cells	↑TIPE	↑cyclin D1, ↑phospho-Rb	[41]
	HT-29 cells	↓TIPE3	↓PI3K-Akt	[31]
	HCT116 cells	↓TIPE	↑p21	[14]
	HCT116 cells	TIPE	-	[42]
Endometrial cancer	Tumor specimens	↑TIPE	↑Ki-67, ↑MMP-9	[43]
ESCC	TE-1, TE-8, and TE-15 cells	↑TIPE	-	[44]
	Eca109 cells	↓TIPE	↑apoptosis	[6]
Gastric cancer	AGS and HGC-27 cells	↑TIPE2	↓Akt, ↓ERK1/2	[45]
	AGS cells xenografted female BALB/c nude mice (4-week-old); Five mice in each group			
	AGS, HGC-27, and SGC-7901 cells	↑TIPE2	↓Akt, ↑GSK3β	[46]
	BGC823 cells	↓TIPE	caspase-3, -8, -9	[47]
	BGC823 cells injected athymic nude mice; four groups containing six animals in each group	↓TIPE	↓tumor growth	
	Tissue samples	↑TIPE	↑metastasis, ↓prognosis	[48]
	AGS, BGC-823, and SGC-7901 cells	TIPE1	↓Wnt/β-catenin, ↓MMP-2, ↓MMP-9	[49]
	BGC-823 cells injected through tail vein into male nude mice (5–6 weeks old), four groups (including control group)			
	MKN-28, SGC-7901, and MGC-803 cells	↑TIPE	-	[50]
	Tissue samples	↑TIPE	-	[51]
	AGS and HGC-27 cells	↑TIPE2	reversal of EMT	[52]
Glioma	U87, U251, and U373 MG cells	↑TIPE2	↓Wnt/β-catenin, ↓EMT	[53]
HCC	Bel7402, SK-Hep-1, HepG2,	↑TIPE	↓YAP phosphorylation	[7]
	SMMC7721 and Huh7 cells			
	Bel7402, SMMC7721, QSG770, HepG2, and HepG2.2.1 cells	TIPE1	↓Rac1	[16]
	Subcutaneously transplanted H22 cells into male BALB/c mice (6–8 weeks old); two groups (including control group) containing at least five mice in each cohort		↓tumor growth and weight	
	HepG2 cells	TIPE2	↓Erk1/2-NF-κB	[27]
Lung cancer	H292 and A549 cells	↑TIPE1	↓cyclin D1, cyclin B1, ↓caspase3,	[15]
			↓caspase 8, ↓MMP-2, ↓MMP-9	
	A549 cells engrafted into the flank of female BALB/c nude mice; two groups (control group and TIPE1 group) containing five mice in each group	↑TIPE1	↓tumor growth	
	H460 and H1299 cells	↑TIPE	↓phosphorylated LATS1	[54]
	A549 cells	↓TIPE	↑p21	[14]
	H1299 cells	TIPE	-	[42]
	Tissue samples	↑TIPE	-	[55]
	H1975 and A549 cells	↑TIPE2	↓Rac1, ↓VEGF	[56]
	Tissue samples	↑TIPE	-	[57]
	H1975 and A549 cells	↑TIPE3	↑Akt, ↑ERK	[30]
	A549 cells transfected into flanks of male BALB/c nude mice (4–6 weeks old), two groups (mock and C-3 flag TIPE3) containing five mice in each group		↑tumor growth	
	NCI-H727 cells	↑TIPE3	-	[31]
	Tissue samples		-	
	NCI-H460 and A549 cells A549 cells injected into flanks of female BALB/c nude mice (4–6 weeks old); two groups (Control and TIPE group) containing 10 mice in each group	↓TIPE	↓MDM2, ↓RAD51 ↓tumor volume	[58]
Melanoma	MDA-MB-435 cells	↓TIPE	↑NR4A1, ↑AP2A1, ↓TOP2A, ↓EGFR ↓PDCL, ↓GTF2F2, ↓IL5, ↓GRAP2, ↓AKAP2, ↓GAP43, ↓ABL1	[9]
NHL	Tissue samples	↑TIPE2	↑prognosis	[59]
	514 NHL patients and 557 cancer-free controls	TIPE	TIPE polymorphism rs1045241C > T	[60]
Osteosarcoma	143b, LM7, HOS, SaOS-2,	↑TIPE	Modulation of miR-138	[61]
	U2OS and MG-63 cells			
	KHOS, 143b, LM7,	↑TIPE	Modulation of miR-99a	[62]
	U2OS and MG-63 cells			
	U2OS cells	↓TIPE	↑p21	[14]
Ovarian cancer	Tissue samples	↑TIPE	↓survival	[63]
	OVCAR-3 cells	↓TIPE	G0/G1 cell cycle arrest, ↑beclin 1, ↑LC II	[64]
	Tissue samples	↑TIPE	-	[65]
Pancreatic cancer	Tissue samples	↑TIPE	↑EGFR	[66]
Prostate cancer	PC-3 cells	↓TIPE	↑IGFBP3, ↑NR4A1, ↑AP2A1, ↓IL5,	[9]
			↓MAPT, ↓TOP2A, ↓TRAF4, ↓EGFR,	
			↓PDCL, ↓GTF2F2, ↓GRAP2, ↓ABL1,	
			↓GAP43, ↓AKAP2, ↓GRIP1	
	PC-3 cells	↑TIPE2	↓PI3K/Akt signaling	[67]
	PC-3 cells	↑TIPE	↑MMPs, ↑VEGFR-2	[68]
	PC-3 cells	TIPE	↑autophagy	[69]
Renal cancer	RCC-RS cells	↑TIPE	-	[39]
Thyroid cancer	Tissue samples	↑TIPE	-	[70]
**Inflammatory diseases**				
Atherosclerosis	*Ldlr*^−/−^ female mice; two groups (wild type and TIPE2^−/−^) containing eight mice in each group	↓TIPE2	↑JNK, ↑NF-κB, ↑p38	[71]
	VSMCs	↓TIPE2	↓contractile proteins, ↑synthetic capacity for growth factors and cytokines	[72]
Colitis	DSS induced male C57BL/6 mice (8-12 weeks old); two groups (wild type and TIPE2^−/−^)	↓TIPE2	↓TNF-α, ↓IL-6, ↓IL-12	[73]
	Colonic epithelial cells	↓TIPE	↑cell death	[74]
	DSS-induced mice (8–10 weeks old); two groups (wild type and TIPE2^−/−^)	↓TIPE	↓survival rate, ↑body weight loss, ↑leukocyte infiltration, ↑bacterial invasion, ↑inflammatory cytokine production in the colon	
Rheumatoid arthritis	AA-FLSs	↑TIPE2	↑DR5, ↑caspase, ↓NF-κB	[75]
	Synovial fibroblasts	↑TIPE2	↓Rac signaling	[76]
**Infectious diseases**				
AIH	PBMCs	↓TIPE2	↑ALT, ↑AST	[77]
Hepatitis B	PBMCs	↓TIPE2	↑perforin, ↑granzyme B, ↑IFN-γ	[78]
	PBMCs	↓TIPE2	↑ALT, ↑AST, ↑total bilirubin	[79]
			↑HBV load	
	Male C57BL/6 mice (10–12 weeks old); two groups (wild type and TIPE2^−/−^)	↓TIPE2	↑hepatic inflammation	
Hepatitis B liver failure	PBMCs	↑TIPE2	↓TNF-α, ↓IL-6	[80]
Hepatitis B	PBMCs	↑TIPE2	↓IL-6, ↓TNF-α, ↓IFN-γ	[81]
Hepatitis-C induced hepatic inflammation	PBMCs	↓TIPE2	↑TLR signaling	[82]
Listeria infection	HEPA1-6 cells	↓TIPE	↑apoptosis, deregulated Rac1-GTP	[83]
Liver fibrosis	HSC-T6 cells	↑TIPE2	↓β-Catenin, ↓cmyc, ↓cyclin D1	[84]
**Neuromuscular and neurodegenerative diseases**				
Myasthenia Gravis	PBMCs	↓TIPE2	↑IL-6, ↑IL-17, ↑IL-21	[85]
Parkinson’s disease	Dopaminergic neurons	↑TIPE1	↑autophagy, ↓mTOR phosphorylation	[86]
**Other diseases**				
CNV	RPE cells	↓TIPE2	↑TNF-α, ↑IL-1β, ↑VEGF	[87]
Diabetes Mellitus	PBMCs	↑TIPE2	↓TNF-α, ↓IL-6	[88]
Diabetic nephropathy	Mesangial cells	↑TIPE	modulation of NADPH oxidase-mediated signaling pathway	[89]
	Male Sprague-Dawley diabetic rats	↑TIPE	-	
Restenosis	VSMCs	↓TIPE2	↑cyclin D1, ↑cyclin D3	[90]
	Male C57BL/6J mice (8–12 weeks old); two groups (wild type and TIPE2^−/−^)	↓TIPE2	↑severity of disease	

## Abbreviations

TIPE or TNFAIP8	Tumor necrosis factor-α-induced protein 8
TIPE1	Tumor necrosis factor-α-induced protein 8-like 1
TIPE2	Tumor necrosis factor-α-induced protein 8-like 2
TIPE3	Tumor necrosis factor-α-induced protein 8-like 3
TLR	Toll-like receptor
TNF-α	Tumor necrosis factor-α
FLIP	Fas-associated death domain-like interleukin-1β-converting enzyme-inhibitory protein
mTOR	Mammalian target of rapamycin
NF-κB	Nuclear factor κ-light-chain-enhancer of activated B cells
MAPK	Mitogen-activated protein kinase
MMP	Matrix metalloproteinase
VEGF	Vascular endothelial growth factor
DLBCL	Diffuse large B-cell lymphoma
EMT	Epithelial-to-mesenchymal transition
EC	Endometrial carcinoma
ESCC	Esophageal squamous cell carcinoma
ERK1/2	Extracellular signal-regulated kinase 1/2
HCC	Hepatocellular carcinoma
NHL	Non-Hodgkin’s lymphoma
PTCL	Peripheral T-cell lymphoma
NSCLC	Non-small cell lung cancer
OS	Osteosarcoma
CNV	Choroidal neovascularization
T2DM	Type 2 diabetes mellitus
MG	Myasthenia gravis
HBV	Hepatitis B virus
HCV	Hepatitis C virus
AIH	Autoimmune hepatitis
AST	Aspartate aminotransferase
ALT	Alanine aminotransferase
PBMCs	Peripheral blood mononuclear cells
DSS	Dextran sodium sulfate
IL-6	Interleukin-6
Ox-LDL	Oxidized low-density lipoprotein
VSMCs	Vascular smooth muscle cells
SM-MHC	Smooth muscle-myosin heavy chain
SmαA	Smooth muscle α-actin
FLSs	Fibroblast-like synoviocytes
AA	Adjuvant arthritis
RPE	Retinal pigment Epithelium

## References

[1] Goldsmith J.R., Chen Y.H. (2017). Regulation of inflammation and tumorigenesis by the TIPE family of phospholipid transfer proteins. Cell. Mol. Immunol..

[2] Shen P., Zhang H., Su Z., Wang S., Xu H. (2015). In Silico Analysis of Tumor Necrosis Factor α-Induced Protein 8-Like-1 (TIPE1) Protein. PLoS ONE.

[3] Kim J.S., Park J., Kim M.S., Ha J.Y., Jang Y.W., Shin D.H., Son J.H. (2017). The Tnfaip8-PE complex is a novel upstream effector in the anti-autophagic action of insulin. Sci. Rep..

[4] Sullivan C., Lage C.R., Yoder J.A., Postlethwait J.H., Kim C.H. (2017). Evolutionary divergence of the vertebrate TNFAIP8 gene family: Applying the spotted gar orthology bridge to understand ohnolog loss in teleosts. PLoS ONE.

[5] Padmavathi G., Banik K., Monisha J., Bordoloi D., Shabnam B., Arfuso F., Sethi G., Fan L., Kunnumakkara A.B. (2018). Novel tumor necrosis factor-α induced protein eight (TNFAIP8/TIPE) family: Functions and downstream targets involved in cancer progression. Cancer Lett..

[6] Sun Z., Liu X., Song J.H., Cheng Y., Liu Y., Jia Y., Meltzer S.J., Wang Z. (2016). TNFAIP8 overexpression: A potential predictor of lymphatic metastatic recurrence in pN0 esophageal squamous cell carcinoma after Ivor Lewis esophagectomy. Tumour Biol..

[7] Dong Q., Fu L., Zhao Y., Xie C., Li Q., Wang E. (2017). TNFAIP8 interacts with LATS1 and promotes aggressiveness through regulation of Hippo pathway in hepatocellular carcinoma. Oncotarget.

[8] Xiao M., Xu Q., Lou C., Qin Y., Ning X., Liu T., Zhao X., Jia S., Huang Y. (2017). Overexpression of TNFAIP8 is associated with tumor aggressiveness and poor prognosis in patients with invasive ductal breast carcinoma. Hum. Pathol..

[9] Day T.F., Mewani R.R., Starr J., Li X., Chakravarty D., Ressom H., Zou X., Eidelman O., Pollard H.B., Srivastava M. (2017). Transcriptome and Proteome Analyses of TNFAIP8 Knockdown Cancer Cells Reveal New Insights into Molecular Determinants of Cell Survival and Tumor Progression. Methods Mol. Biol..

[10] Yu B., Xu L., Cai M., Zhang D., Li S. (2018). Effect of tumor necrosis factor-α-induced protein 8 on the immune response of CD4+ T lymphocytes in mice following acute insult. Mol. Med. Rep..

[11] Kumar D., Whiteside T.L., Kasid U. (2000). Identification of a novel tumor necrosis factor-α-inducible gene, SCC-S2, containing the consensus sequence of a death effector domain of fas-associated death domain-like interleukin-1β-converting enzyme-inhibitory protein. J. Biol. Chem..

[12] Xin Y., Wan D.H., Wang X., Gao X.J., Xu X.J., Ju X.L., Li A.M. (2016). Effect of tumor necrosis factor-induced protein 8 on T-cell-mediated immunity in mice after thermal injury. J. Biol. Regulat. Homeostat. Agents.

[13] Luan Y.Y., Yao Y.M., Sheng Z.Y. (2013). The tumor necrosis factor-α-induced protein 8 family in immune homeostasis and inflammatory cancer diseases. J. Biol. Regulat. Homeostat. Agents.

[14] Lowe J.M., Nguyen T.A., Grimm S.A., Gabor K.A., Peddada S.D., Li L., Anderson C.W., Resnick M.A., Menendez D., Fessler M.B. (2017). The novel p53 target TNFAIP8 variant 2 is increased in cancer and offsets p53-dependent tumor suppression. Cell Death Differ..

[15] Wu X., Ma Y., Cheng J., Li X., Zheng H., Jiang L., Zhou R. (2017). TIPE1 function as a prognosis predictor and negative regulator of lung cancer. Oncotarget.

[16] Zhang Z., Liang X., Gao L., Ma H., Liu X., Pan Y., Yan W., Shan H., Wang Z., Chen Y.H. (2015). TIPE1 induces apoptosis by negatively regulating Rac1 activation in hepatocellular carcinoma cells. Oncogene.

[17] Zhang L., Liu R., Luan Y.Y., Yao Y.M. (2018). Tumor Necrosis Factor-α Induced Protein 8: Pathophysiology, Clinical Significance, and Regulatory Mechanism. Int. J. Biol. Sci..

[18] Luan Y.Y., Yao Y.M., Zhang L., Dong N., Zhang Q.H., Yu Y., Sheng Z.Y. (2011). Expression of tumor necrosis factor-α induced protein 8 like-2 contributes to the immunosuppressive property of CD4(+)CD25(+) regulatory T cells in mice. Mol. Immunol..

[19] Li Z., Jia W., Niu J., Zhang L. (2018). Understanding the roles of negative immune regulator TIPE2 in different diseases and tumourigenesis. Histol. Histopathol..

[20] Ruan Q., Wang P., Wang T., Qi J., Wei M., Wang S., Fan T., Johnson D., Wan X., Shi W. (2014). MicroRNA-21 regulates T-cell apoptosis by directly targeting the tumor suppressor gene Tipe2. Cell Death Dis..

[21] Zhang G., Hao C., Lou Y., Xi W., Wang X., Wang Y., Qu Z., Guo C., Chen Y., Zhang Y. (2010). Tissue-specific expression of TIPE2 provides insights into its function. Mol. Immunol..

[22] Freundt E.C., Bidere N., Lenardo M.J. (2008). A different TIPE of immune homeostasis. Cell.

[23] Sun H., Gong S., Carmody R.J., Hilliard A., Li L., Sun J., Kong L., Xu L., Hilliard B., Hu S. (2008). TIPE2, a negative regulator of innate and adaptive immunity that maintains immune homeostasis. Cell.

[24] Zhang L., Shi Y., Wang Y., Zhu F., Wang Q., Ma C., Chen Y.H., Zhang L. (2011). The unique expression profile of human TIPE2 suggests new functions beyond its role in immune regulation. Mol. Immunol..

[25] Lou Y., Zhang G., Geng M., Zhang W., Cui J., Liu S. (2014). TIPE2 negatively regulates inflammation by switching arginine metabolism from nitric oxide synthase to arginase. PLoS ONE.

[26] Shao Y., Chen H., Lv M., Li C., Zhang W., Li Y., Zhao X., Bao Y. (2017). A novel TNFAIP8 gene mediates l-arginine metabolism in *Apostichopus japonicus*. Fish Shellfish Immunol..

[27] Zhang Y.H., Yan H.Q., Wang F., Wang Y.Y., Jiang Y.N., Wang Y.N., Gao F.G. (2015). TIPE2 inhibits TNF-α-induced hepatocellular carcinoma cell metastasis via Erk1/2 downregulation and NF-κB activation. Int. J. Oncol..

[28] Fayngerts S.A., Wang Z., Zamani A., Sun H., Boggs A.E., Porturas T.P., Xie W., Lin M., Cathopoulis T., Goldsmith J.R. (2017). Direction of leukocyte polarization and migration by the phosphoinositide-transfer protein TIPE2. Nat. Immunol..

[29] Li T., Wang W., Gong S., Sun H., Zhang H., Yang A.G., Chen Y.H., Li X. (2018). Genome-wide analysis reveals TNFAIP8L2 as an immune checkpoint regulator of inflammation and metabolism. Mol. Immunol..

[30] Wang G., Guo C., Zhao H., Pan Z., Zhu F., Zhang L., Wang Q. (2018). TIPE3 differentially modulates proliferation and migration of human non-small-cell lung cancer cells via distinct subcellular location. BMC Cancer.

[31] Fayngerts S.A., Wu J., Oxley C.L., Liu X., Vourekas A., Cathopoulis T., Wang Z., Cui J., Liu S., Sun H. (2014). TIPE3 is the transfer protein of lipid second messengers that promote cancer. Cancer Cell.

[32] Lian K., Ma C., Hao C., Li Y., Zhang N., Chen Y.H., Liu S. (2017). TIPE3 protein promotes breast cancer metastasis through activating AKT and NF-κB signaling pathways. Oncotarget.

[33] Lee D., Kim M.S., Park J., Jhon G.J., Son J.H., Shin D.H. (2014). A preliminary X-ray study of murine Tnfaip8/Oxi-α. Int. J. Mol. Sci..

[34] Zhang X., Wang J., Fan C., Li H., Sun H., Gong S., Chen Y.H., Shi Y. (2009). Crystal structure of TIPE2 provides insights into immune homeostasis. Nat. Struct. Mol. Biol..

[35] Antony P., Baby B., Vijayan R. (2016). Molecular insights into the binding of phosphoinositides to the TH domain region of TIPE proteins. J. Mol. Model..

[36] Wang K., Ren Y., Liu Y., Zhang J., He J.J. (2017). Tumor Necrosis Factor (TNF)-α-Induced Protein 8-like-2 (TIPE2) Inhibits Proliferation and Tumorigenesis in Breast Cancer Cells. Oncol. Res..

[37] Garcia J.A., Ferreira H.L., Vieira F.V., Gameiro R., Andrade A.L., Eugenio F.R., Flores E.F., Cardoso T.C. (2017). Tumour necrosis factor-α-induced protein 8 (TNFAIP8) expression associated with cell survival and death in cancer cell lines infected with canine distemper virus. Vet. Comp. Oncol..

[38] Zhang C., Chakravarty D., Sakabe I., Mewani R.R., Boudreau H.E., Kumar D., Ahmad I., Kasid U.N. (2006). Role of SCC-S2 in experimental metastasis and modulation of VEGFR-2, MMP-1, and MMP-9 expression. Mol. Ther..

[39] Kumar D., Gokhale P., Broustas C., Chakravarty D., Ahmad I., Kasid U. (2004). Expression of SCC-S2, an antiapoptotic molecule, correlates with enhanced proliferation and tumorigenicity of MDA-MB 435 cells. Oncogene.

[40] Shi T.Y., Cheng X., Yu K.D., Sun M.H., Shao Z.M., Wang M.Y., Zhu M.L., He J., Li Q.X., Chen X.J. (2013). Functional variants in TNFAIP8 associated with cervical cancer susceptibility and clinical outcomes. Carcinogenesis.

[41] Miao Z., Zhao T., Wang Z., Xu Y., Song Y., Wu J., Xu H. (2012). SCC-S2 is overexpressed in colon cancers and regulates cell proliferation. Tumour Biol..

[42] Monteith J.A., Mellert H., Sammons M.A., Kuswanto L.A., Sykes S.M., Resnick-Silverman L., Manfredi J.J., Berger S.L., McMahon S.B. (2016). A rare DNA contact mutation in cancer confers p53 gain-of-function and tumor cell survival via TNFAIP8 induction. Mol. Oncol..

[43] Liu T., Gao H., Yang M., Zhao T., Liu Y., Lou G. (2014). Correlation of TNFAIP8 overexpression with the proliferation, metastasis, and disease-free survival in endometrial cancer. Tumour Biol..

[44] Hadisaputri Y.E., Miyazaki T., Suzuki S., Yokobori T., Kobayashi T., Tanaka N., Inose T., Sohda M., Kuwano H. (2012). TNFAIP8 overexpression: Clinical relevance to esophageal squamous cell carcinoma. Ann. Surg. Oncol..

[45] Zhu Y., Tao M., Wu J., Meng Y., Xu C., Tian Y., Zhou X., Xiang J., Zhang H., Xie Y. (2016). Adenovirus-directed expression of TIPE2 suppresses gastric cancer growth via induction of apoptosis and inhibition of AKT and ERK1/2 signaling. Cancer Gene Ther..

[46] Wu J., Zhang H., Xu C., Xu H., Zhou X., Xie Y., Tao M. (2016). TIPE2 functions as a metastasis suppressor via negatively regulating β-catenin through activating GSK3β in gastric cancer. Int. J. Oncol..

[47] Hu R., Qiu X., Hong S., Meng L., Hong X., Qiu J., Yang J., Zhuang G., Liu Z. (2016). Clinical significance of TIPE expression in gastric carcinoma. OncoTargets Ther..

[48] Chen L., Yang X., Yang X., Fan K., Xiao P., Zhang J., Wang X. (2016). Association between the expression levels of tumor necrosis factor-α-induced protein 8 and the prognosis of patients with gastric adenocarcinoma. Exp. Ther. Med..

[49] Liu W., Chen Y., Xie H., Guo Y., Ren D., Li Y., Jing X., Li D., Wang X., Zhao M. (2018). TIPE1 suppresses invasion and migration through down-regulating Wnt/β-catenin pathway in gastric cancer. J. Cell. Mol. Med..

[50] Li Y., Jing C., Chen Y., Wang J., Zhou M., Liu X., Sun D., Mu L., Li L., Guo X. (2015). Expression of tumor necrosis factor α-induced protein 8 is upregulated in human gastric cancer and regulates cell proliferation, invasion and migration. Mol. Med. Rep..

[51] Yang M., Zhao Q., Wang X., Liu T., Yao G., Lou C., Zhang Y. (2014). TNFAIP8 overexpression is associated with lymph node metastasis and poor prognosis in intestinal-type gastric adenocarcinoma. Histopathology.

[52] Yin H., Huang X., Tao M., Hu Q., Qiu J., Chen W., Wu J., Xie Y. (2017). Adenovirus-mediated TIPE2 overexpression inhibits gastric cancer metastasis via reversal of epithelial-mesenchymal transition. Cancer Gene Ther..

[53] Liu Z.J., Liu H.L., Zhou H.C., Wang G.C. (2016). TIPE2 Inhibits Hypoxia-Induced Wnt/β-Catenin Pathway Activation and EMT in Glioma Cells. Oncol. Res..

[54] Han Y., Tang Z., Zhao Y., Li Q., Wang E. (2018). TNFAIP8 regulates Hippo pathway through interacting with LATS1 to promote cell proliferation and invasion in lung cancer. Mol. Carcinogenesis.

[55] Wang L., Song Y., Men X. (2014). Variance of TNFAIP8 expression between tumor tissues and tumor-infiltrating CD4+ and CD8+ T cells in non-small cell lung cancer. Tumour Biol..

[56] Li Z., Guo C., Liu X., Zhou C., Zhu F., Wang X., Wang Q., Shi Y., Wang J., Zhao W. (2016). TIPE2 suppresses angiogenesis and non-small cell lung cancer (NSCLC) invasiveness via inhibiting Rac1 activation and VEGF expression. Oncotarget.

[57] Dong Q.Z., Zhao Y., Liu Y., Wang Y., Zhang P.X., Jiang G.Y., Dong X.J., Cui Q.Z., Wang E.H. (2010). Overexpression of SCC-S2 correlates with lymph node metastasis and poor prognosis in patients with non-small-cell lung cancer. Cancer Sci..

[58] Xing Y., Liu Y., Liu T., Meng Q., Lu H., Liu W., Hu J., Li C., Cao M., Yan S. (2018). TNFAIP8 promotes the proliferation and cisplatin chemoresistance of non-small cell lung cancer through MDM2/p53 pathway. Cell Commun. Signal..

[59] Hao C., Zhang N., Geng M., Ren Q., Li Y., Wang Y., Chen Y.H., Liu S. (2016). Clinical Significance of TIPE2 Protein Upregulation in Non-Hodgkin’s Lymphoma. J. Histochem. Cytochem..

[60] Zhang Y., Wang M.Y., He J., Wang J.C., Yang Y.J., Jin L., Chen Z.Y., Ma X.J., Sun M.H., Xia K.Q. (2012). Tumor necrosis factor-α induced protein 8 polymorphism and risk of non-Hodgkin’s lymphoma in a Chinese population: A case-control study. PLoS ONE.

[61] Zhou Z., Li Z., Shen Y., Chen T. (2017). MicroRNA-138 directly targets TNFAIP8 and acts as a tumor suppressor in osteosarcoma. Exp. Ther. Med..

[62] Xing B., Ren C. (2016). Tumor-suppressive miR-99a inhibits cell proliferation via targeting of TNFAIP8 in osteosarcoma cells. Am. J. Transl. Res..

[63] Liu T., Gao H., Chen X., Lou G., Gu L., Yang M., Xia B., Yin H. (2013). TNFAIP8 as a predictor of metastasis and a novel prognostic biomarker in patients with epithelial ovarian cancer. Br. J. Cancer.

[64] Wang J., Gao H., Liu G., Gu L., Yang C., Zhang F., Liu T. (2018). Tumor necrosis factor α-induced protein 8 expression as a predictor of prognosis and resistance in patients with advanced ovarian cancer treated with neoadjuvant chemotherapy. Hum. Pathol..

[65] Liu T., Xia B., Lu Y., Xu Y., Lou G. (2014). TNFAIP8 overexpression is associated with platinum resistance in epithelial ovarian cancers with optimal cytoreduction. Hum. Pathol..

[66] Liu K., Qin C.K., Wang Z.Y., Liu S.X., Cui X.P., Zhang D.Y. (2012). Expression of tumor necrosis factor-α-induced protein 8 in pancreas tissues and its correlation with epithelial growth factor receptor levels. Asian Pac. J. Cancer Prev..

[67] Lu Q., Liu Z., Li Z., Chen J., Liao Z., Wu W.R., Li Y.W. (2016). TIPE2 Overexpression Suppresses the Proliferation, Migration, and Invasion in Prostate Cancer Cells by Inhibiting PI3K/Akt Signaling Pathway. Oncol. Res..

[68] Zhang C., Kallakury B.V., Ross J.S., Mewani R.R., Sheehan C.E., Sakabe I., Luta G., Kumar D., Yadavalli S., Starr J. (2013). The significance of TNFAIP8 in prostate cancer response to radiation and docetaxel and disease recurrence. Int. J. Cancer.

[69] Niture S., Ramalinga M., Kedir H., Patacsil D., Niture S.S., Li J., Mani H., Suy S., Collins S., Kumar D. (2018). TNFAIP8 promotes prostate cancer cell survival by inducing autophagy. Oncotarget.

[70] Duan D., Zhu Y.Q., Guan L.L., Wang J. (2014). Upregulation of SCC-S2 in immune cells and tumor tissues of papillary thyroid carcinoma. Tumour Biol..

[71] Lou Y., Liu S., Zhang C., Zhang G., Li J., Ni M., An G., Dong M., Liu X., Zhu F. (2013). Enhanced atherosclerosis in TIPE2-deficient mice is associated with increased macrophage responses to oxidized low-density lipoprotein. J. Immunol..

[72] Zhang G., Zhang W., Lou Y., Xi W., Cui J., Geng M., Zhu F., Chen Y.H., Liu S. (2013). TIPE2 deficiency accelerates neointima formation by downregulating smooth muscle cell differentiation. Cell Cycle.

[73] Lou Y., Sun H., Morrissey S., Porturas T., Liu S., Hua X., Chen Y.H. (2014). Critical roles of TIPE2 protein in murine experimental colitis. J. Immunol..

[74] Sun H., Lou Y., Porturas T., Morrissey S., Luo G., Qi J., Ruan Q., Shi S., Chen Y.H. (2015). Exacerbated experimental colitis in TNFAIP8-deficient mice. J. Immunol..

[75] Shi C., Zhang S., Hong S., Pang J., Yesibulati Y., Yin P., Zhuang G. (2016). The pro-apoptotic effects of TIPE2 on AA rat fibroblast-like synoviocytes via regulation of the DR5-caspase-NF-κB pathway in vitro. OncoTargets Ther..

[76] Shi C., Wang Y., Zhuang G., Qi Z., Li Y., Yin P. (2017). Tumor necrosis factor-α-induced protein8 like 2 regulates lipopolysaccharideinduced rat rheumatoid arthritis immune responses and is associated with Rac activation and interferon regulatory factor 3 phosphorylation. Mol. Med. Rep..

[77] Qian J., Meng Z., Guan J., Zhang Z., Wang Y. (2017). Expression and roles of TIPE2 in autoimmune hepatitis. Exp. Ther. Med..

[78] Zhang W., Zhang J., Zhao L., Shao J., Cui J., Guo C., Zhu F., Chen Y.H., Liu S. (2015). TIPE2 protein negatively regulates HBV-specific CD8(+) T lymphocyte functions in humans. Mol. Immunol..

[79] Xi W., Hu Y., Liu Y., Zhang J., Wang L., Lou Y., Qu Z., Cui J., Zhang G., Liang X. (2011). Roles of TIPE2 in hepatitis B virus-induced hepatic inflammation in humans and mice. Mol. Immunol..

[80] Wang L.Y., Fan Y.C., Zhao J., Gao S., Sun F.K., Han J., Yang Y., Wang K. (2014). Elevated expression of tumour necrosis factor-α-induced protein 8 (TNFAIP8)-like 2 mRNA in peripheral blood mononuclear cells is associated with disease progression of acute-on-chronic hepatitis B liver failure. J. Viral Hepat..

[81] Fan Y.C., Zhang Y.Y., Wang N., Sun Y.Y., Wang K. (2017). Tumor necrosis factor-α-induced protein 8-like 2 (TIPE2) is associated with immune phases of patients with chronic hepatitis B. Oncotarget.

[82] Kong L., Liu K., Zhang Y.Z., Jin M., Wu B.R., Wang W.Z., Li W., Nan Y.M., Chen Y.H. (2013). Downregulation of TIPE2 mRNA expression in peripheral blood mononuclear cells from patients with chronic hepatitis C. Hepatol. Int..

[83] Porturas T.P., Sun H., Buchlis G., Lou Y., Liang X., Cathopoulis T., Fayngerts S., Johnson D.S., Wang Z., Chen Y.H. (2015). Crucial roles of TNFAIP8 protein in regulating apoptosis and Listeria infection. J. Immunol..

[84] Xu D.D., Li X.F., Li Y.H., Liu Y.H., Huang C., Meng X.M., Li J. (2018). TIPE2 attenuates liver fibrosis by reversing the activated hepatic stellate cells. Biochem. Biophys. Res. Commun..

[85] Zhang Y., Shao Z., Zhang X., Jia X., Xia Y., Zhang Y., Xin N., Guo M., Chen J., Zheng S. (2015). TIPE2 Play a Negative Role in TLR4-Mediated Autoimmune T Helper 17 Cell Responses in Patients with Myasthenia Gravis. J. Neuroimmune Pharmacol..

[86] Ha J.Y., Kim J.S., Kang Y.H., Bok E., Kim Y.S., Son J.H. (2014). Tnfaip8 l1/Oxi-β binds to FBXW5, increasing autophagy through activation of TSC2 in a Parkinson’s disease model. J. Neurochem..

[87] Suo L.G., Cui Y.Y., Bai Y., Qin X.J. (2016). Anti-inflammatory TIPE2 inhibits angiogenic VEGF in retinal pigment epithelium. Mol. Immunol..

[88] Liu Y., Wang X., Zhao Y., Zhao P., Wang L., Zhai Q., Zhang X., Tian W., Xiang X., Li T. (2017). Upregulation of Tumor Necrosis Factor-α-Induced Protein 8-Like 2 mRNA Is Negatively Correlated with Serum Concentrations of Tumor Necrosis Factor-α and Interleukin 6 in Type 2 Diabetes Mellitus. J. Diabetes Res..

[89] Zhang S., Zhang Y., Wei X., Zhen J., Wang Z., Li M., Miao W., Ding H., Du P., Zhang W. (2010). Expression and regulation of a novel identified TNFAIP8 family is associated with diabetic nephropathy. Biochim. Biophys. Acta.

[90] Zhang G., Zhao L., Wang Y., Shao J., Cui J., Lou Y., Geng M., Zhang N., Chen Y.H., Liu S. (2015). TIPE2 protein prevents injury-induced restenosis in mice. Biochim. Biophys. Acta.

[91] Bordoloi D., Roy N.K., Monisha J., Padmavathi G., Kunnumakkara A.B. (2016). Multi-Targeted Agents in Cancer Cell Chemosensitization: What We Learnt from Curcumin Thus Far. Recent Patents Anti-Cancer Drug Discov..

[92] Kunnumakkara A.B., Bordoloi D., Harsha C., Banik K., Gupta S.C., Aggarwal B.B. (2017). Curcumin mediates anticancer effects by modulating multiple cell signaling pathways. Clin. Sci..

[93] Aletaha D., Neogi T., Silman A.J., Funovits J., Felson D.T., Bingham C.O., Birnbaum N.S., Burmester G.R., Bykerk V.P., Cohen M.D. (2010). Rheumatoid arthritis classification criteria: An American College of Rheumatology/European League against Rheumatism collaborative initiative. Arthritis Rheumat..

[94] Kouchaki E., Daneshvar Kakhaki R., Tamtaji O.R., Dadgostar E., Behnam M., Zaribaf A., Nikoueinejad H., Akbari H., Asemi Z. (2018). Correlation of serum levels and gene expression of tumor necrosis factor-α-induced protein-8 like-2 with Parkinson disease severity. Metab. Brain Dis..

